# Ultra‐high performance supercritical fluid chromatography coupled to tandem mass spectrometry for antidoping analyses: Assessment of the inter‐laboratory reproducibility with urine samples

**DOI:** 10.1002/ansa.202000131

**Published:** 2020-12-05

**Authors:** Gioacchino Luca Losacco, Marco Rentsch, Kateřina Plachká, Fabrice Monteau, Emmanuelle Bichon, Bruno Le Bizec, Lucie Nováková, Raul Nicoli, Tiia Kuuranne, Jean‐Luc Veuthey, Davy Guillarme

**Affiliations:** ^1^ School of Pharmaceutical Sciences University of Geneva CMU – Rue Michel‐Servet 1 Geneva 4 1211 Switzerland; ^2^ Institute of Pharmaceutical Sciences of Western Switzerland University of Geneva CMU – Rue Michel‐Servet 1 Geneva 1211 Switzerland; ^3^ Waters AG Taefernstrasse 14a Baden‐Daetwill 5405 Switzerland; ^4^ Department of Analytical Chemistry Faculty of Pharmacy in Hradec Králové Charles University Heyrovského 1203 Hradec Králové 500 05 Czech Republic; ^5^ LABERCA Oniris, INRAE Nantes F‐44307 France; ^6^ Swiss Laboratory for Doping Analyses University Center of Legal Medicine Lausanne‐Geneva, Centre Hospitalier Universitaire Vaudois, University of Lausanne Chemin des Croisettes 22 Epalinges 1066 Switzerland

**Keywords:** anti‐doping analyses, inter‐laboratory reproducibility, tandem mass spectrometry, ultra high‐performance supercritical fluid chromatography

## Abstract

The aim of this study was to assess the interlaboratory reproducibility of ultra‐high performance supercritical fluid chromatography coupled with tandem mass spectrometry method for routine antidoping analyses. To do so, a set of 21 doping agents, spiked in urine and analyzed after dilute and shoot treatment, was used to assess the variability of their retention times between four different laboratories, all equipped with the same chromatographic system and with the same ultra‐high performance supercritical fluid chromatography stationary phase chemistry. The average relative standard deviations (RSD%) demonstrated a good reproducibility of the retention times for 19 out of 21 analytes, with RSD% values below 3.0%. Only for two substances, namely fenbutrazate and niketamide, the retention was not repeatable between laboratories, with RSD% of approximately 15% in both cases. This behaviour was associated with (a) the low organic modifier percentage (around 2‐4%) in the mobile phase at the corresponding retention times, and (b) the influence of the system volume on poorly retained analytes. An analysis on seven “blind” urines was subsequently carried out in the same four laboratories. In these blind samples, either one, two, or none of the 21 doping agents previously analyzed were present at an unknown concentration. Each laboratory had to perform the identification of the compounds in the samples and estimate their concentrations. All laboratories assigned all target analytes correctly in all blind urine samples and provide a comparable estimation of their concentrations.

## 1 INTRODUCTION

The world of antidoping analyses is in constant evolution, as more strict criteria are regularly defined by the World Anti‐Doping Agency (WADA) to promote clean sport and enhance the deterrence from doping practices. Therefore, a lot of emphases is put on the improvement of analytical techniques already employed in routine anti‐doping laboratories.^1^, ^2^, ^3^ Moreover, new analytical approaches are also considered, with the potential of additional advantages to the analysts such as faster time analysis and improved throughput. In this context, the implementation of ultra‐high performance supercritical fluid chromatography, coupled to tandem mass spectrometry (UHPSFC‐MS/MS), has received a great deal of interest in the last few years from different research groups and antidoping laboratories throughout the world.^4^, ^5^, ^6^, ^7^, ^8^ Thanks to its unique separation profile, complementary to that achievable with reversed‐phase liquid chromatography (RPLC) and excellent kinetic performance due to the use of supercritical fluid in the mobile phase, UHPSFC‐MS/MS can indeed be successfully employed with challenging samples, providing similar or even better results than ultra‐high‐performance liquid chromatography (UHPLC) coupled to MS/MS systems.^9^, ^10^, ^11^ Furthermore, UHPSFC‐MS/MS does not pose the same challenges related to sample preparation as gas chromatography (GC), as it does not require any derivatization step prior to analysis. One of the historical issues with SFC was its scarce method robustness.^12^, ^13^, ^14^ The use of mobile phases with limited percentages of cosolvent (<10‐15%), in combination with the limited capability of the pumping system in handling supercritical fluids, has often translated, in the past, into poorly robust methods. This was due to the high compressibility of the mobile phase itself, as well as from the formation of density gradients throughout the column. Moreover, the instrumentation was not able to perform rugged analyses and quantitative performance was always poor. However, the shift towards UHPSFC, allowed by the introduction of a new generation of instruments in 2012, seems to have successfully adressed this challenge. In the past 3‐5 years, there has been an increasing number of studies focusing on how UHPSFC can guarantee similar performance to UHPLC in terms of method robustness. The comparison has been demonstrated with standard compounds and, more recently, with biological matrices too.^8^, ^15^, ^16^ Nonetheless, nothing has been done so far in assessing the robustness of a UHPSFC bioanalytical method across different laboratories. This point is of vital importance if UHPSFC has to be considered a viable option for routine analyses in regulated environments.

The aim of this work was to assess the retention times variability of a UHPSFC‐MS/MS method across four different laboratories, using a set of 21 doping agents spiked, at two different concentration levels, in urine treated following the dilute‐and‐shoot (DS) procedure. Secondly, an evaluation of the performance of such UHPSFC‐MS/MS method in analyzing seven different blind urine samples in the same four laboratories was carried out.

## 2 MATERIALS AND METHODS

### 2.1 Chemicals and reagents

Reference doping agents, namely amiloride, amphetamine, atenolol, cocaine metabolite (benzoylecgonine), fenbutrazate, fentanyl, fentanyl metabolite (norfentanyl), fluoxymesterone, gestrinone, hydrochlorothiazide, JWH 250 metabolite (JWH‐N‐(5‐carboxypentyl)), niketamide, niketamide metabolite (N‐ethylnicotinamide), prednisone, probenecide, propranolol, salbutamol, stanozolol, tamoxifene, terbutaline, trimetazidine, and one internal standard (salbutamol*‐d5*) were kindly provided by the Swiss Laboratory for Doping Analyses (Epalinges, Switzerland).

The minimal quality level for solvents and reagents in each laboratory were: methanol (MeOH), acetonitrile (ACN), and water (H_2_O) of LC‐MS grade and ammonium formate (AmF) at 99.9% purity level. Pressurized carbon dioxide (CO_2_) of at least 3.0 grade (99.9%) was employed in each laboratory.

### 2.2 Standard solutions and biological samples treatment

Stock solutions of each doping agent were prepared in MeOH at a concentration of 1 mg/mL. From these solutions, two stock solutions containing all 21 doping agents at either 500 or 50 ng/mL and 50 or 5 ng/mL were prepared in ACN/H_2_O 75:25 v/v (Table S1).

Urine samples have been prepared using a dilute‐and‐shoot (DS) procedure, with a mixture of ACN/H2O 75:25 *v/v* as sample diluent. The choice of this solvent was based on a previous work.^9^ The DS procedure is as follows: six urine samples, obtained from three healthy men and three healthy women volunteers, were mixed to obtain a representative urine sample pool. The pooled urine was filtered through a 0.22 µm nylon membrane. Subsequently, two aliquots of 100 µL of filtered pooled urine each were taken and spiked with 100 µL of stock solution at 500–50 ng/mL or 50–5 ng/mL and 800 µL of sample diluent solvent, to have quality control (QC) urine samples at two levels of concentration.

Blind urines were obtained from the Swiss Laboratory for Doping Analyses and prepared according to the DS sample treatment procedure. Dilutions of either 10 or 100 times from original samples with ACN/H_2_O 75:25 v/v were performed before injection (Table S2) to obtain comparable signal intensities among the samples.

All samples have been prepared at the Swiss Laboratory for Doping Analyses and kept at a temperature of –22°C. Ready‐to‐inject vials containing negative quality control samples (blanks), positive control samples (QC), and blind urines were sent to all participating laboratories.

### 2.3 UHPSFC‐MS/MS instrumentation and chromatographic conditions

Each laboratory performed the analyses on a Waters Acquity UPC^2^ system (Waters, Milford, MA, USA), equipped with a binary solvent delivery pump (BSM), an autosampler (SM), a column oven (CM), a UV detector (PDA) fitted with an 8 µL flow‐cell and a two‐step (active + passive) backpressure regulator (BPR). Each chromatographic system was hyphenated to a tandem mass spectrometer (triple quadrupole ‐ QqQ; Table S3), equipped with an electrospray ionization (ESI) source. The hyphenation between the chromatographic and tandem MS systems was made via a dedicated double‐T splitter interface from Waters. Additional make‐up solvent (pure MeOH at a flow‐rate of 0.3 mL/min) was brought by a Waters Isocratic Solvent Manager (ISM) pump.

Each MS/MS instrument operated in positive and negative ESI modes, using Selected Reaction Monitoring (SRM) as the acquisition mode. Polarity switching between the two ionization modes was performed within the same injection. Source temperature was set at 140°C. Nitrogen was used as the desolvation gas at 900 L/h and 450°C and as the cone gas at either 100 or 150 L/h. Argon was chosen as the collision gas. The capillary voltage was set at ± 1.0 kV. Individual values for transitions, cone voltages and collision energies, as well as the ionization mode have been listed in Table S4 of the Supplementary material. Dwell times were set at 20 ms for ESI positive, while a dwell time of 25 ms was chosen for the ESI negative.

The column employed by all laboratories was the Torus 2‐PIC 100 × 3.0 mm ID 1.7 µm (Waters, Milford, MA, USA). The organic cosolvent was a mixture of MeOH/H_2_O 98/2 v/v + 20 mM AmF. More information regarding the chromatographic method and the stationary phase choice can be found in.^8^ Two Torus 2‐PIC columns have been employed in this study. Data acquisition and instrument control were performed with MassLynx v4.1 or 4.2 (Waters, Milford, MA, USA), while data processing was performed with TargetLynx v4.1 (Waters, Milford, MA, USA).

### 2.4 Sequence structure and data treatment

**FIGURE 1:**
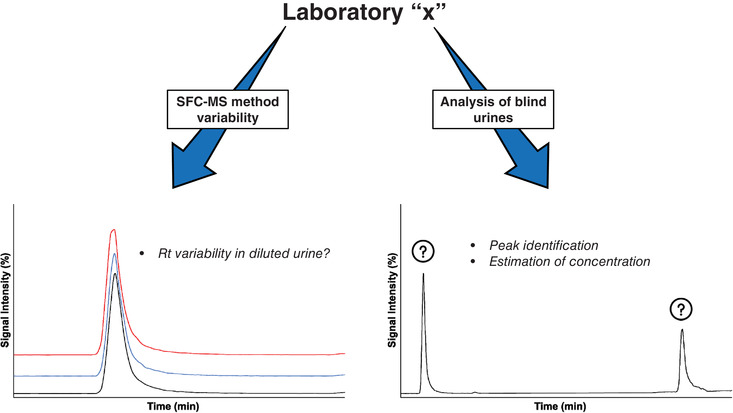
Description of the workflow employed in each laboratory participating in the study.

For the purpose of assessing the interlaboratory reproducibility, the sample sequence used by all laboratories was identical. Each laboratory was asked to perform a test of the UHPSFC‐MS/MS method robustness using the QC samples (Figure 1). Subsequently, an identification and concentration estimation of potential doping agents in seven blind urines was carried out (Figure 1). A specific injection sequence was systematically applied in each laboratory: after multiple injections of a blank urine on the column, the two QC urine samples were injected in triplicate; then, the seven blind urines were also tested, with a double blank injection in between. Finally, the QC urine samples were once again injected in triplicate.

To assess the inter‐laboratory retention times variability of the set of doping agents, the retention times obtained for all compounds present in each QC sample, injected at the beginning and end of the sequence, have been used to perform the calculations. The total variance ($s_{T}^{2}$) was obtained using the following formula:

(1) $$s_{T}^{2}=\;s_{r}^{2}+\;s_{s}^{2}+\;s_{l}^{2}$$

where $s_{r}^{2}$ represents the variance obtained between the injections of each triplicate analysis, $s_{s}^{2}$ represents the variability between the triplicate's injections at the beginning and end of the sequence and $s_{l}^{2}$ is the variance between laboratories. From the values of $s_{T}^{2}$ obtained for each compound, the relative standard deviation (RSD) was calculated to assess the inter‐laboratory reproducibility. RSD values, represented in %, were plotted in a violin plot created using Plotly Chart Studio (https://chart-studio.plot.ly). Data treatment was performed via Microsoft Excel 2019.

## 3 RESULTS AND DISCUSSION

### 3.1 Interlaboratory reproducibility

In a previous study revolving on the robustness assessment of a UHPSFC‐MS/MS method for routine antidoping analyses, the combination of the latest generation UHPSFC stationary phases and the use of water as an additive in the mobile phase was successfully used to achieve an excellent stability of the analytical method.^8^ The study, however, was carried out entirely in the same laboratory, raising still questions related to the potential inter‐laboratory repeatability. This point must be properly assessed before UHPSFC can be considered as a viable alternative in routine laboratories. Only one work focusing on assessing the inter‐laboratory reproducibility for a UHPSFC method was made, using a UV detector and simple pharmaceutical formulation.^15^ In the present work, the robustness of a generic UHPSFC method using complex matrices (biological fluids), as well as an MS detector (MS/MS) hyphenated to the UHPSFC instrument was investigated.

**FIGURE 2:**
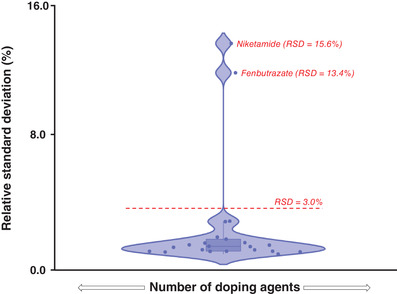
Violin plot representing the relative standard deviation (RSD%) values, relative to the inter‐laboratory variability of retention times, obtained for the doping agents.

**FIGURE 3:**
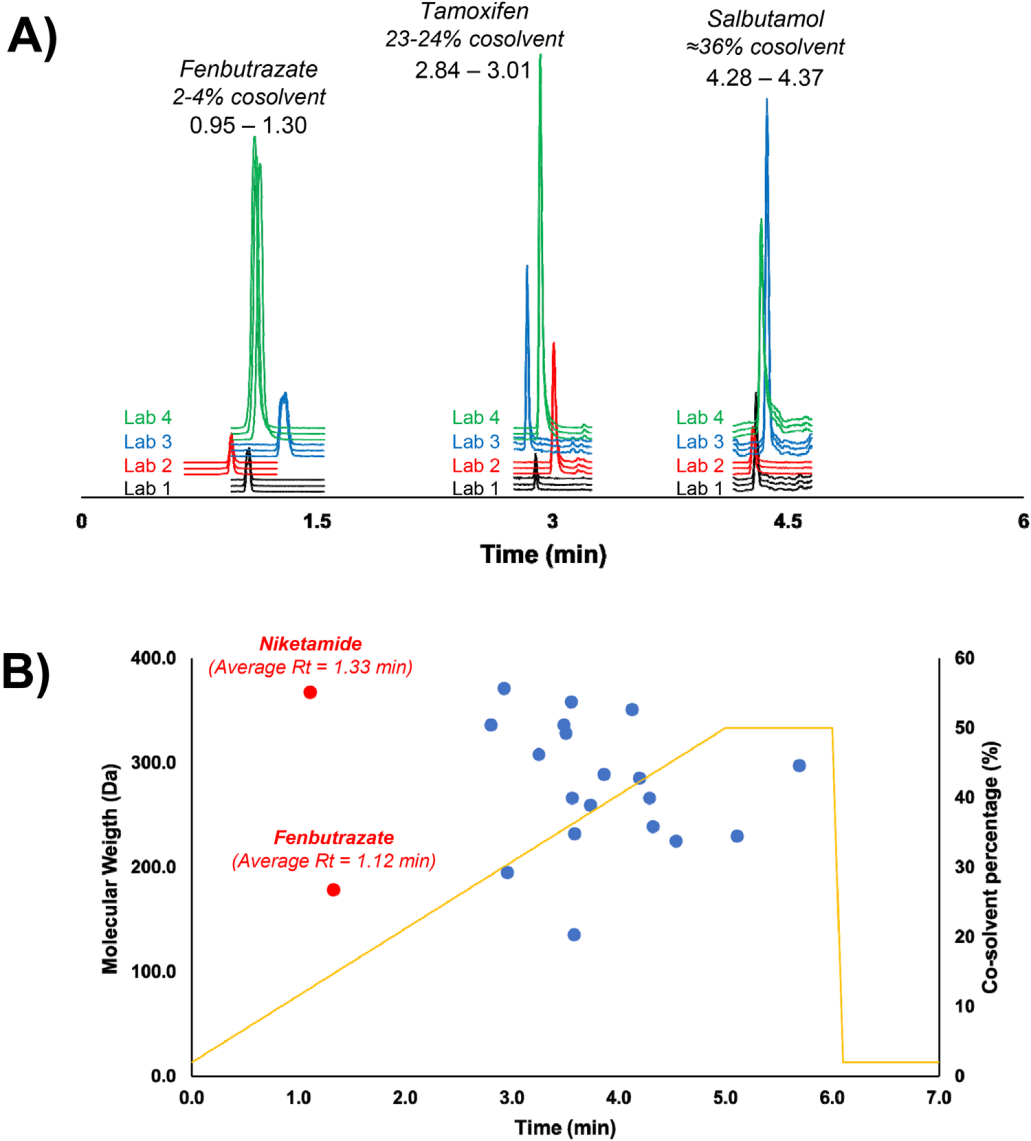
A) Overlay of the chromatograms for three compounds (fenbutrazate, tamoxifen, and salbutamol) obtained by each laboratory; B) Plot of the average retention times, obtained by the four laboratories, for the 21 doping agents across the gradient profile used in this study.

This inter‐laboratory evaluation of retention times variability was assessed for the 21 doping agents. Relative standard deviations (RSD), representing the variability of the retention times of each target compound found in each laboratory expressed in percentage, have been calculated and represented in a violin plot (Figure 2). Among the 21 analytes, 19 offer a suitable retention time repeatability between the four laboratories, with RSD (%) values below 3.0%. Only two compounds, namely niketamide and fenbutrazate, have shown a significant variability, as indicated by their position in the violin plot (Figure 2). Average RSD (%) values related to the intra‐injection variability for these two analytes are also relatively high when compared to the other compounds (Table S5). To better investigate the reasons for this poor retention reproducibility, a correlation with the gradient conditions was made. In Figure 3A, the corresponding chromatograms of fenbutrazate, tamoxifen, and salbutamol were plotted. An immediate trend is visible from these three doping agents: a higher percentage of organic cosolvent in the mobile phase induces a better reproducibility of the retention times. The impact is further highlighted in Figure 3B: among the 21 doping agents, 19 of them elute quite late along with the chosen gradient profile (t_r_ > 2.7 min), while niketamide and fenbutrazate do not interact strongly with the stationary phase (no H‐bond donor group on the structures) and are poorly retained. When these two compounds are eluted, the mobile phase is mostly under its supercritical state, due to the low percentage of cosolvent employed (between 2% and 4%). Under such conditions, the total backpressure of the system and mobile phase temperature can strongly impact retention.^17^, ^18^ This phenomenon, well‐known in UHPSFC, is extremely hard to control and therefore, may have an important impact on the early‐eluting compounds. In the present study, the small differences in the UHPSFC‐MS system setup (ie, tubing dimensions, presence of switching valves, etc.; Table S3) and slight differences in pressure between 2‐PIC columns, generate some differences in total system pressure between laboratories and could explain the variability of early‐eluting compounds. Besides the pressure differences between the UHPSFC systems used in different laboratories, the system extracolumn volume might also cause further variability in the retention profile, especially for early‐eluting compounds. The UHPSFC system employed by all laboratories (Waters Acquity UPC^2^) has already been characterized as an instrument with an important extracolumn volume.^19^ This additional volume translates into an increase of the retention times, especially for early‐eluting compounds. The changes in the retention times highly depend on the system setup, therefore even minimal changes in the extracolumn volume of the UHPSFC system can translate into an important variability for those analytes who do not interact well with the stationary phase. Due to the low retention times for niketamide and fenbutrazate, a variation of their retention can have a higher impact on the calculation of their variability compared to those analytes with high retention times. Moreover, the two Torus 2‐PIC columns employed do not belong to the same batch, which might potentially have also contributed to the variability seen for these two analytes among the four laboratories. Finally, from Figure 3A, it is possible to also see a peak‐splitting phenomenon for fenbutrazate, another common issue with early‐eluting compounds.

Regardless of the unsatisfactory results found for niketamide and fenbutrazate, the variability of retention times was low for the majority of compounds when analyzed in the four laboratories. The employment of important percentages of the liquid organic modifier contributed to the overall method robustness. More importantly, as already mentioned in,^8^ the use of water as a mobile phase additive, together with the use of the latest generation of UHPSFC stationary phase (Torus 2‐PIC), cause a substantial improvement in retention reproducibility under UHPSFC conditions. While in^8^ this statement was verified only in one laboratory, this work confirms that such method robustness was maintained between different laboratories.

### 3.2 Analysis of blind urine samples

**FIGURE 4:**
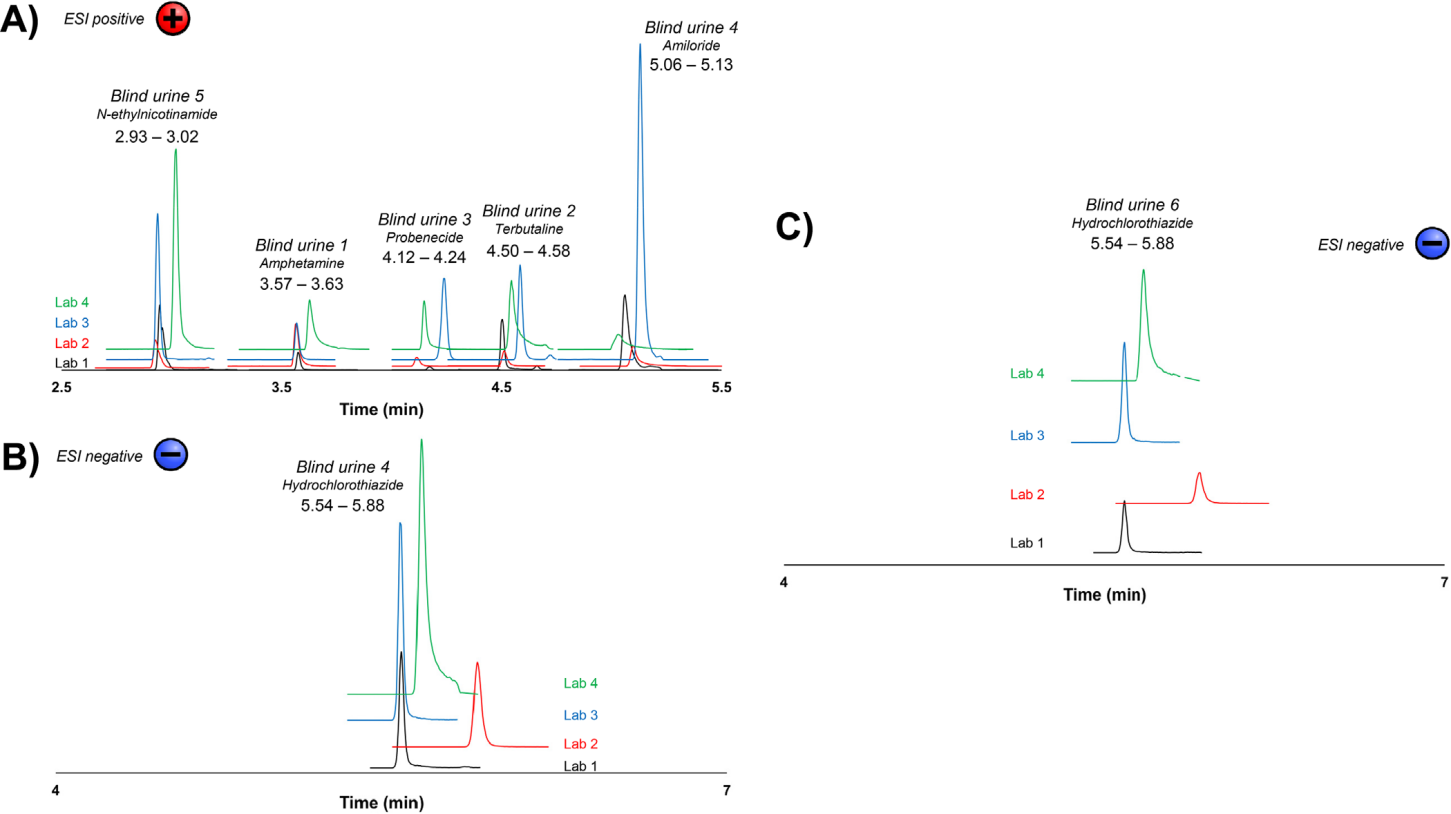
A) Chromatograms of each analyte (N‐ethylnicotinamide, amphetamine, probenecide, terbutaline, and amiloride) present in blind urine samples 1 to 5 using ESI positive mode by all four laboratories; B) Chromatograms of hydrochlorothiazide found in blind urine 4 using ESI negative mode, obtained by all four laboratories; C) Chromatograms of hydrochlorothiazide found in blind urine 6 using ESI negative mode, obtained by all four laboratories

Following the evaluation of the inter‐laboratory reproducibility, the performance of the developed UHPSFC‐MS/MS method was assessed, by analyzing a set of seven different urine samples, each containing zero to two of the previously discussed 21 target compounds. These excretion urine samples were called “blind urines” and were used to demonstrate the fitness of the method for routine anti‐doping analysis purposes. Each laboratory performed the analysis without knowing which, and how many analytes were present in each blind urine, nor their concentration. The aim was, quite simply, to verify how all laboratories were capable to properly perform a routine screening for urine samples, consisting of the identification step, as well as a rather simple estimation of the concentration of the doping agents. In Figure 4, the chromatograms of the different compounds present in six blind urines are represented. Blind urine 7 did not contain any doping agent, while two doping agents (amiloride and hydrochlorothiazide) were present in blind urine 4. First, all doping agents were successfully identified by all laboratories; the window range in retention time between the four laboratories was kept to a minimum, with five of the six compounds eluting within a window of 0.12 min on the maximum (Figure 4A). Hydrochlorothiazide, present in blind urines 4 and 6 (Figure 4B,C), is the only sample presenting extended elution window (0.34 min), due to the shift in the retention profile witnessed by laboratory 2, although the calculated RSD was only equal to 2.8%.

Having assessed the identification step, the focus shifted toward the estimation of the concentration for each analyte. By using the same doping agents spiked in the quality control samples at two different concentration levels, a two‐point calibration curve for each identified analyte was made. Due to the limited number of QC samples, only a simple estimation of each unknown analyte was possible. The curves were, then, used to estimate the concentrations of each doping agent found in the seven blind urines. The estimated concentrations, described in Table 1, have been compared to the respective Minimum Required Performance Levels (MRPL) values for each doping agent. No values from blind urine 7 have been shown, as no substances were present in this sample. The results found by the four laboratories were overall consistent for all analytes present in the six blind urines (Table 1), illustrating the potential of UHPSFC‐MS/MS during the screening process in anti‐doping analyses. Although some differences, regarding the estimated concentrations, were seen (Table 1), it should be noted that the MS/MS systems used by the four laboratories were not identical, although they consisted of the same MS analyzer type (triple quadrupole). This difference in the UHPSFC‐MS/MS configuration could explain the differences in the estimations, especially if a possible saturation of the signal intensity occurred. Regardless of the differences observed between some laboratories, the UHPSFC‐MS/MS method was capable of giving the same results, when considering the relative MRPL values.

**TABLE 1:** Estimated concentration values of all found analytes in blind urine samples 1 to 6 from all four laboratories, compared against their related MRPL levels

Estimated concentration (ng/mL)						
	Blind urine 1 (Amphetamine)	Blind urine 2 (Terbutaline)	Blind urine 3 (Probenecide)	Blind urine 4 (Amiloride + Hydrochlorothiazide)	Blind urine 5 (Niketamide metabolite)	Blind urine 6 (Hydrochlorothiazide)
**Laboratory 1**	89	8	408	26 246	22	54
**Laboratory 2**	69	8	463	39 481	24	77
**Laboratory 3**	81	10	461	36 323	21	71
**Laboratory 4**	109	8	408	36 796	25	151
***MRPL***	*100*	*20*	*200*	*200*	*100*	*200*

## 4 CONCLUSIONS

In this work, an assessment of the interlaboratory reproducibility of a UHPSFC‐MS/MS method between four laboratories has been made on a set of 21 doping agents spiked in a biological matrix. The results showed acceptable robustness of the method, with a low variability of the retention times for 19 out of 21 analytes. This was associated with the employment of an important amount of organic cosolvent in the mobile phase. For two early‐eluting compounds, nikethamide and fenbutrazate, the observed variabilities were higher, indicating that in future method development and inclusion of new compounds in the method, there might be potential issues in terms of retention times reproducibility for those compounds that elute with a limited amount of organic cosolvent in the mobile phase. Moreover, the influence of the instrument volume should not be neglected for such target analytes, as it might negatively impact the reproducibility of their retention times, too.

In the second part of this article, the analysis of a set of seven blind urine samples was performed. Each laboratory has successfully identified the unknown analytes present in blind urines. Moreover, the estimation of the concentrations for each unknown doping agents performed across the four laboratories gave consistent results overall, when compared to the respective MRPL values. These findings indicate, therefore, that UHPSFC‐MS/MS has managed, in these years, to evolve into a technique which could be potentially employed for screening procedure in antidoping laboratories.

## Supporting information

Supporting information
