# Supplementary material for: Ultra‐high performance supercritical fluid chromatography coupled to tandem mass spectrometry for antidoping analyses: Assessment of the inter‐laboratory reproducibility with urine samples
Source: Anal Sci Adv. 2020 Dec 5;2(1-2):68–75. doi: 10.1002/ansa.202000131 (PMC10989559; doi:10.1002/ansa.202000131)
Supplement: Supplementary file 1 — Supporting information [file ANSA-2-68-s001.docx]

**Supplementary material for:**

**Ultra-high performance supercritical fluid chromatography coupled to tandem mass spectrometry for antidoping analyses: assessment of the inter-laboratory reproducibility with urine samples.**

*AUTHORS****:*** *Gioacchino Luca LOSACCO^(1,2)^, Marco RENTSCH^(3)^, Kateřina PLACHKÁ^(4)^, Fabrice MONTEAU^(5)^, Emmanuelle BICHON^(5)^, Bruno LE BIZEC^(5)^, Lucie NOVÁKOVÁ^(4)^, Raul NICOLI^(6)^, Tiia KUURANNE^(6)^, Jean-Luc VEUTHEY^(1,2)^, Davy GUILLARME^(1,2)^*

(1) School of Pharmaceutical Sciences, University of Geneva, CMU – Rue Michel-Servet 1, 1211 Geneva 4, Switzerland.

(2) Institute of Pharmaceutical Sciences of Western Switzerland, University of Geneva, CMU – Rue Michel-Servet 1, 1211 Geneva 4, Switzerland.

(3) Waters AG, Taefernstrasse 4, 5405 Baden-Daetwill, Switzerland.

(4) Department of Analytical Chemistry, Faculty of Pharmacy in Hradec Králové, Charles University, Heyrovského 1203, 500 05 Hradec Králové, Czech Republic.

(5) LABERCA, Oniris, INRAE, F-44307 Nantes, France.

(6) Swiss Laboratory for Doping Analyses, University Center of Legal Medicine Lausanne-Geneva, Centre Hospitalier Universitaire Vaudois, University of Lausanne, Chemin des Croisettes 22, 1066 Epalinges, Switzerland.

**Table S1**: Stock solutions of each doping agents at two different concentration levels.

| **Name** | **Concentration – lower (ng.mL^-1^)** | **Concentration – higher (ng.mL^-1^)** |
| --- | --- | --- |
| Amiloride | 5 | 50 |
| Amphetamine | 50 | 500 |
| Atenolol | 5 | 50 |
| Benzoylecgonine | 50 | 500 |
| Fenbrutazate | 5 | 50 |
| Fentanyl | 5 | 50 |
| Fluoxymesterone | 50 | 500 |
| Gestrinone | 5 | 50 |
| Hydrochlorothiazide | 50 | 500 |
| JWH 205 met 1 | 5 | 50 |
| Niketamide | 5 | 50 |
| Niketamide met | 5 | 50 |
| Norfentanyl | 5 | 50 |
| Prednisone | 5 | 50 |
| Probenecide | 50 | 500 |
| Propanolol | 5 | 50 |
| Salbutamol | 5 | 50 |
| *Salbutamol-d5 (ISTD)* | *5* | *50* |
| Stanozolol | 5 | 50 |
| Tamoxifene | 5 | 50 |
| Terbutaline | 5 | 50 |
| Trimetazidine | 5 | 50 |

**Table S2**: Dilution steps performed for each blind urine from original samples.

| **Sample** | **Dilution step** |
| --- | --- |
| Blind urine 1 | Dilution x100 |
| Blind urine 2 | Dilution x10 |
| Blind urine 3 | Dilution x100 |
| Blind urine 4 | Dilution x10 |
| Blind urine 5 | Dilution x10 |
| Blind urine 6 | Dilution x10 |
| Blind urine 7 | Dilution x10 |

**Table S3**: Systems set-up used in this study.

| **Chromatographic system** | **Tandem mass spectrometer** |
| --- | --- |
| Waters Acquity UPC^2^ (BSM+SM+BPR+8-positions CM+PDA) | Waters Xevo TQ-S |
| Waters Acquity UPC^2^ (BSM+SM+BPR+2-positions CM+PDA) | Waters Xevo TQ-XS |
| Waters Acquity UPC^2^ (BSM+SM+BPR+2-positions CM+PDA) | Waters Xevo microTQ-S |
| Waters Acquity UPC^2^ (BSM+SM+BPR+2-positions CM+PDA) | Waters Xevo TQ-S |

**Table S4**: MS/MS parameters for each doping agent.

| **Name** | **ESI mode** | **Precursor (m/z)** | **Product (m/z)** | **Cone voltage (V)** | **Collision energy (eV)** |
| --- | --- | --- | --- | --- | --- |
| Amiloride | POS | 230 | 171 | 10 | 18 |
| Amphetamine | POS | 136 | 91 | 25 | 14 |
| Atenolol | POS | 267 | 145 | 48 | 28 |
| Benzoylecgonine | POS | 290 | 105 | 10 | 30 |
| Fenbrutazate | POS | 368 | 191 | 20 | 22 |
| Fentanyl | POS | 337 | 188 | 38 | 20 |
| Fluoxymesterone | POS | 337 | 281 | 42 | 20 |
| Gestrinone | POS | 309 | 241 | 42 | 24 |
| Hydrochlorothiazide | NEG | 296 | 269 | 50 | 20 |
| JWH 205 met 1 | POS | 366 | 121 | 50 | 20 |
| Niketamide | POS | 179 | 108 | 44 | 18 |
| Niketamide met | POS | 151 | 80 | 40 | 20 |
| Norfentanyl | POS | 233 | 84 | 30 | 18 |
| Prednisone | POS | 359 | 313 | 30 | 12 |
| Probenecide | NEG | 284 | 140 | 25 | 24 |
| Propanolol | POS | 260 | 116 | 24 | 20 |
| Salbutamol | POS | 240 | 148 | 24 | 16 |
| Stanozolol | POS | 329 | 81 | 52 | 40 |
| Tamoxifene | POS | 372 | 72 | 25 | 25 |
| Terbutaline | POS | 226 | 152 | 20 | 16 |
| Trimetazidine | POS | 267 | 181 | 36 | 10 |
| *Salbutamol-d5 (ISTD)* | POS - NEG | 243 – 241 | 151 – 149 | 12 | 16 |

**Table S5:** Average RSD (%) values for intra-injection variability recorded by each laboratory.

| **Name** | **Lab 1** | **Lab 2** | **Lab 3** | **Lab 4** | **Average all labs** |
| --- | --- | --- | --- | --- | --- |
| Amiloride | 0.03% | 0.00% | 0.09% | 0.00% | 0.03% |
| Amphetamine | 0.00% | 0.00% | 0.00% | 0.03% | 0.01% |
| Atenolol | 0.07% | 0.03% | 0.07% | 0.02% | 0.05% |
| Benzoylecgonine | 0.04% | 0.00% | 0.00% | 0.03% | 0.02% |
| Fenbrutazate | 0.00% | 0.33% | 0.41% | 0.75% | 0.37% |
| Fentanyl | 0.00% | 0.00% | 0.16% | 0.05% | 0.05% |
| Fluoxymesterone | 0.04% | 0.04% | 0.00% | 0.07% | 0.04% |
| Gestrinone | 0.04% | 0.00% | 0.04% | 0.06% | 0.04% |
| Hydrochlorothiazide | 0.02% | 0.03% | 0.00% | 0.04% | 0.02% |
| JWH 205 met 1 | 0.00% | 0.04% | 0.14% | 0.04% | 0.05% |
| Niketamide | 0.39% | 0.09% | 0.33% | 0.67% | 0.37% |
| Niketamide met | 0.00% | 0.05% | 0.00% | 0.02% | 0.02% |
| Norfentanyl | 0.00% | 0.08% | 0.00% | 0.00% | 0.02% |
| Prednisone | 0.00% | 0.04% | 0.08% | 0.08% | 0.05% |
| Probenecide | 0.04% | 0.07% | 0.07% | 0.05% | 0.05% |
| Propanolol | 0.00% | 0.00% | 0.12% | 0.03% | 0.04% |
| Salbutamol | 0.00% | 0.03% | 0.00% | 0.02% | 0.01% |
| Stanozolol | 0.04% | 0.12% | 0.08% | 0.00% | 0.06% |
| Tamoxifene | 0.00% | 0.00% | 0.00% | 0.00% | 0.00% |
| Terbutaline | 0.00% | 0.03% | 0.00% | 0.00% | 0.01% |
| Trimetazidine | 0.00% | 0.00% | 0.12% | 0.00% | 0.03% |
| *Salbutamol-d5 (ISTD)* | *0.00%* | *0.03%* | *0.03%* | *0.00%* | *0.02%* |
